# Ecological Cognitive Analysis of Chinese Harmonious Discourse

**DOI:** 10.3389/fpsyg.2021.713809

**Published:** 2021-11-18

**Authors:** Zou Chun-ling

**Affiliations:** Department of Foreign Languages, Harbin University of Science and Technology, Harbin, China

**Keywords:** Chinese culture, eco-cognition, Chinese discourse, dialectical philosophy, Chinese harmonious discourse, ecological cognitive mechanism, dialectical opposite-unity philosophy

## Abstract

This study is concerned with cognitively and consciously enacting a new dialectical opposite-unity approach into Chinese harmonious discourse (CHD) analysis in an ecological perspective, which contributed to converting antagonistic thinking between human and nature into an ecological harmonious one cultivated into an unconscious state. The method applied is primarily the theoretical analysis and interpretation, due to the newness of this subject and the lack of corpus data. The motivation of this paper is evoked by the discovery of various cognition dissonances and insufficiencies with the academic development of newly born ecolinguistics. On a micro or specified level, this paper presents a cutting-edge example of an ecologically cognitive approach to the analysis of CHD, based on Chinese dialectical opposite-unity philosophy, to construct a higher-level cognition mechanism into a habitually unconscious thinking state. Such a mechanism has its practical significance in devoting to alleviating the ecological crisis by a change in ways of thinking, mediating cognitive dissonance brought about by the crisis, and improving the one-sided cognition deficiency brought about by ways of antagonistic thinking in order to maintain the ecological harmony. The theoretical significance lies in it demonstrating the cognitive process about how the unconscious ecological harmony cognition is cultivated by the conscious operational opposite-unity cognition procedure, with the ultimate purpose to achieve and maintain a real ecological harmony, under the cross-cultural background.

## Introduction

With the COVID-19 pandemic breaking out globally in early 2020, the problem of ecological crisis has been studying in domains of linguistics, social sciences, psychology, and discourse analysis, etc. Then, countless studies have warned of the disastrous consequences of such a crisis and put forth proposals for global sustainability (e.g., Lenton et al., 2020; Vuong, 2021a). All scholars have been striving to build a new ecology-oriented core value in their respective cultures, it is necessary to incorporate insights from different cultures and disciplines. The study of Quan-Hoang Vuong proposed a solution to the problem of environmental crisis in the form of a new core cultural value centered around environmental protection, in order to enrich and improve the so-called “the eco-deficit culture” (Vuong, 2021b, p. 285), being an important reference for this article.

This article proceeded to assume that the fundamental nature of destructive discourses from the ecological crisis comes from the antagonistic ways of cognition between humans and nature, definitely resulting in “the eco-deficit culture.” Therefore, this article intends to solve the above thinking problem by emphasizing insights from Chinese culture, philosophy, ecology, cognition, which could shed new light on our reshaping, refreshing the multidisciplinary research of ecological beneficial discourse and promoting its role in our long-term quest to protect ecology, so as to reshape human behaviors and ways of thinking.

This article adopts a Chinese culture and philosophy-based dialectical opposite-unity (DOU) cognition mechanism, which echoes the assertion of Chinese philosopher Laozi, founder of Taoism, who advocates doing nothing that goes against nature. Furthermore, DOU has been seeking to find efficient cultural and cognitive responses to the nowadays ecological destruction and sustainability threats, in order to negotiate the antagonistic cognition and raise human thinking to a higher and harmonious level for protecting nature. Therefore, DOU represents a seemingly cognitive unity and unconsciousness, from thousands of years conscious cultivating the extraction ability of life laws toward Chinese various opposite-unity discourses.

### The Motivation of Eco-Cognition

This paper offers a tentative interpretation of how a DOU cognitive mechanism is developed and used, which fell within the ecologically beneficial approach. Well-spoken or well-written passages can evoke our deepest emotions and elicit all manner of consciousness and could reactions. This is usually taken to be an insurmountable explanatory challenge for ecological approaches to cognitive science (Steffensen, 2018, p. 1), termed as ecological cognition (eco-cognition). In order to propose such an interdisciplinary “ecology + cognition interactivity-based” approach to the analysis of Chinese harmonious discourse (CHD) meaning, this paper presents a cutting-edge example of an ecological cognitive approach to discourse analysis, which is rarely studied throughout ecolinguistics.

By proposing the typical Chinese philosophy of Confucianism, Taoism, and Mohism (CTM), commonly characterized by DOU thinking mechanism as the basis of ecological cognition, this paper attempted to analyze and interpret CHD in an interdisciplinary ecological cognitive perspective, for supplementing discourse theories, and for promoting ecological harmony.

On the one hand, in order to raise an ecological harmonious ethics awareness and self-realization in a deep sense, the concept of “eco-cognition” with an ecologically beneficial goal and with DOU as the mechanism could give an impulse to reconsider harmonious discourse research as a unique and consistent mode of telling ecological stories. Therefore, one of the motivations of the eco-cognition mechanism is concerned with interpreting CHD on a micro and specified level instead of a macro and general one.

On the other hand, some scholars believe that ecolinguistics leads to a new “holistic” worldview, in which “everything is inter-connected, inter-dependent and inter-acting,” looking at ecolinguistics as a dialectical philosophy” (Døør and Madsen, 2007, p. 268). This is an evident marker indicating the research inclination to a dialectical interaction in the ecological study, echoing Chinese DOU philosophy and cognition, which is the other motivation.

To sum up, as philosophically minded ecolinguists look more profoundly at discourse study on a meta-level, the ecological cognitive problem is unavoidable.

### The Etymology of Eco-Cognition

Ecolinguistics, as a transdisciplinary science (or a dialectical philosophy), transcends traditional linguistics and creates an awareness of the interdependency of all things and ideas (Finke, 2014). In this perspective, the prefix “eco” has its far-reaching implication into a dialectical philosophy of interaction and harmony, which echoes the typical Chinese assertion of “dialectical opposite-unity” philosophy from CTM. As such a new dialectical ecological approach is presented, so does a new cognitive approach to the discourse analysis nestling with it, embodying the concept of reconstructing the cognition mechanism into the natural ecosystem.

With the ecological turn, ecocriticism (Garrard, 2014), ecopoetics (Knickerbocker, 2012), ecofeminism (Adams and Gruen, 2014), ecopsychology (Fisher, 2013), ecosociology (Stevens, 2012), political ecology (Robbins et al., 2012), and environmental communication (Cox, 2012) also presented themselves, but no one deals with eco-cognition. Besides the above reason, the terminology rationality of “eco-cognition” is still in that one is reminded that the prefix “eco-” has become increasingly attached to all sorts of descriptors, including eco-tourism, eco-vehicle, eco-houses, and eco-lifestyles, as well as, in this process, has acquired a large number of meanings (Mühlhäusler, 2018, p. 136).

On this level, “eco-” is regarded as a philosophical perspective guiding and improving various research including linguistics and cognition, functioning as the core concept and essential connotation of “eco” disciplines, according to which “eco” is also rationally taken as a philosophical guideline for the cognition research. Additionally, traditional Chinese cognition is always based on philosophy, so the term “eco-cognition” is always a “philosophy + cognition” integration, a Chinese “DOU” philosophy-based cognitive mechanism, specifically. As eco-linguistics has been inviting many analytical perspectives into a nexus or established theoretical core, eco-cognition is one of them with its dialectical approach as the harmonious solution for cognitive dissonance brought about by the one-sided positive discourse or critical discourse.

Thus, “eco-” has become a commonly used prefix to manifest the interdisciplinary and philosophical stance, such as the term “eco-literacy” coined by David Orr and Fritjof Capra for awareness of ecological problems and the role language plays in creating this awareness (Orr, 1992; Capra, 1995). And the terminology of “ecocognition” in this paper is subsequently and rationally coined as the analytical perspective, developing into a higher “DOU” cognitive mechanism used to solve cognitive dissonance exemplified in CHD, in order to cognitively achieve and maintain the ecological harmony in the world. Rather, it belongs to a particular Taoism mode of description and interpretation that draws a higher, subtle, and abstract generalization or emergent property across the phenomenological experience of many language users.

### The Function of Eco-Cognition

**FIGURE 1:**
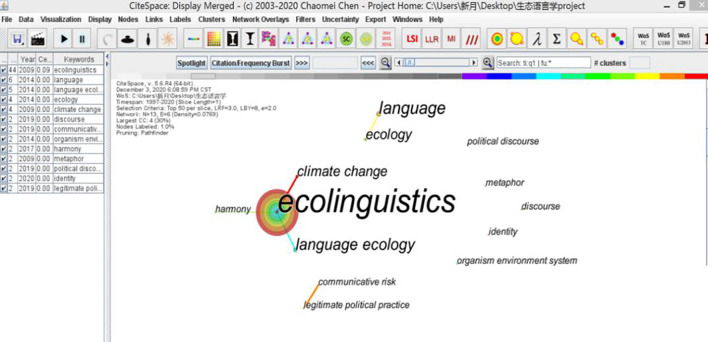
Multiword key terms output by CiteSpace based on 74 journals on ecolinguistics 1997–2020.

As mentioned in Figure 1, eco-cognition functions primarily as a way to balance the cognitive dissonance in order to achieve ecological harmony both in mind and discourse. The state of cognitive dissonance occurs when people believe that two of their psychological representations are inconsistent with each other. More formally, a pair of cognitions is inconsistent if one cognition follows from the obverse (opposite) of the other (Cooper, 2007, p. 6), which is roughly presented almost throughout Chinese discourses. The inconsistent cognitions are primarily formulated as opposite lexicons and represented as opposite concepts, both as an approach of telling Chinese Taoism. What the eco-cognition can function is to integrate the two opposites into a harmonious unity, that is, a more subtle and abstract construal.

In order to fully understand these opposite units, scholars invite the concepts of “ecologically dialectical philosophy” (Døør and Bang, 1996, p. 19), “deep ecology” (Naess, 1995), and “identity” (Leibniz, 1969), but no one is quite appropriate to illustrate Chinese way of opposite-unity thinking. Chinese harmonious “unity” is an acceptable way of achieving higher-level cognition, Taoism. Pursuing such a higher level cognition has always been the main concern throughout the history of Chinese philosophy, with “dialectical opposite-unity” as the mechanism and operational process, it is traditionally addressed as a yin + yang philosophy of nature, proposed by Chinese Laozi, according to which the research of principles/theories/framework of ethics, behaviors, and discourse are cooperatively developing.

Such a traditional Chinese approach also has its role in mediating the cognitive dissonance and antagonistic thinking characterized by formulation inconsistency, into an ecological harmonious state characterized by unity and consistency. It is an accomplishment not only needs consciously cognitive efforts, but also the appropriate and effective strategies and theories, different from the conventional unconscious, basic, and embodied cognition. In this perspective, not only the structure, characteristics, and purposes of harmonious discourse should be illustrated, also the corresponding cognitive mechanism.

## Significances and Objectives of This Paper

In order to make clear the research objectives of this paper, some discourse problems to be solved must be first reviewed.

### Literature Review

Ecolinguistics is generally divided into two categories: Haugen’s metaphorical model and Halliday’s non-metaphorical model. “Ecology,” “environment,” “language ecology,” “ecological language,” and “ecologically critical/positive discourse” become the basic concepts of ecological linguistics. Scholars have been developing roughly four strands that differ in how they interpret what the environment of language is. These strands include symbolic ecology, natural ecology, socio-cultural ecology, and cognitive ecology (Steffensen and Fill, 2014), the last of which echoes the assertions of this paper.

For a more accurate literature review, an advanced search was carried out in Web of Science (WOS) with “ecolinguistics” as the keyword, source category as WOS core collection, and literature type as articles in 74 related journals were searched, and the “Keywords” of 74 journals were analyzed by CiteSpace software. Time: 1997–2020.

**FIGURE 2:**
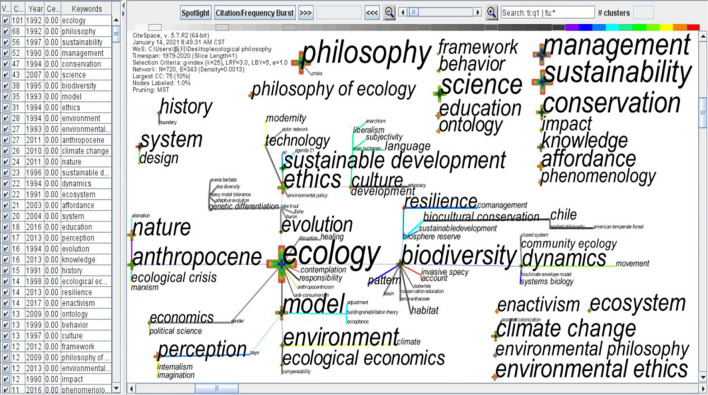
Multiword key terms output by CiteSpace based on 1077 articles on ecological philosophy, 1976–2020.

As mentioned in Figure 2, from the above CiteSpace literature, a relatively complete study of dialectical ecological cognition (eco-cognition) of harmonious discourse was ignored, although Marxist and post-Foucauldian “dialectical-relational” critical approach was preliminarily postulated (Fairclough, 2014). The present ecological discourse pieces of research have been developing in two directions:

(1) ecologically critical discourse analysis (e.g., Haugen, 1972; Fairclough, 1989; Halliday, 1990; Finke, 2014; Van Dijk, 2015);
(2) ecologically positive discourse analysis (e.g., Martin, 1999, 2004; Dunayer, 2001; Macgilchrist, 2007);
(3) Both of the two with finding and correcting human-centered ideology resulted in the global ecological crisis as the main concern.

Chinese ecolinguistics is a newly born multidisciplinary research project, beginning from the founding of the Centre for Ecolinguistics at South China Agricultural University initiated by Huang Guowen. This is the first international conference on ecolinguistics in China (November 25–27, 2016, South China Agricultural University, Guangzhou). From then on, ecolinguistics is taken more as a philosophy and a state of mind in which harmony, above all other ideas, is dominant.

On the macro-level, the research of “harmony” invites Chinese linguists to profoundly probe the ecologically beneficial discourse, termed as harmonious discourse. CHD mainly revolves around the balance of the above two discourse categories by way of Chinese DOU philosophy and cognition.

On the micro-level, the majority of Chinese scholars follow Halliday’s systemic-functional way in dealing with ecolinguistics (e.g., Huang, 2017; Wenjuan, 2017; Wang, 2019; He, 2020), in a macro and general way. This paper looks for other micro ways to approach ecolinguistics, especially CHD, integrating cognitive ecology into a single coherent DOU framework, in a micro and specified way. Therefore, there exist disciplinary and perspective differences between the author and other Chinese scholars, by insisting on a cognitive explanatory mechanism to CHD.

Ecolinguistics is supposed to be seen as a unified ecological worldview (cf. Wenjuan, 2017). More work on this can be expected in the next few decades. For this reason, the work of the author in ecological cognitive science has sought to re-describe DOU mechanism associated with discourse meaning as a variety of, or at least as continuous with, Chinese ecological philosophy, with the spirit of CTM as the backbone.

Our conclusion is that the only way to coherently relate ecological and cognitive conceptions of CHD meaning is to understand the latter as a Chinese philosophy-dependent identification of a hugely heterogeneously opposite class of formulations with a unifying property (unity).

### Research Objectives

Ecological meanings of discourses are inspired by models of cognition as fundamentally interactive (Gibson, 1979), and such an inevitable ecological cognitive interconnection gives rise to the recent work of cognitive linguistics presenting a more or less ecological turn (e.g., Zlatev and Blomberg, 2016). The ecological cognitive mechanism, such as the transfer and construal of ideas, urgently needs Chinese DOU as the explanatory framework. Therefore, the research objectives of this paper were as follows:

(1) On a macro-level, the interpretation of the CHD in an ecological cognitive perspective;
(2) on a meso-level, the illustration of the origin, definition, characteristics, and goals of a newly born ecological cognition in a newly born CHD;
(3) on a micro-level, the explanation of the CHD by Chinese “dialectical opposite-unity” ecological cognitive mechanism, in order to solve some cognitive dissonance and improve the basic cognition to a higher level.

### Research Significance

The most important contribution made by the ecological cognitive approach is that it accounts adequately for the role played by dynamic harmonious discourse patterns in the control of higher-level opposite-unity cognition.

On the one hand, the theoretical significance of this paper is that it did not only contribute to establishing a new relationship between humans and nature but also to the self-improvement of cognitive theories, mechanisms, models, and values onto a higher level.

On the other hand, the practical significance of this paper is that it examined the DOU mechanism of CHD from the perspective of ecological cognition, which was conducive to providing a rational path for improving the effectiveness of understanding a newly born harmonious discourse, under the cross-cultural background. Such a cognition up-gradation is expected to reduce ecological crisis and achieve real ecological harmony, to some degree.

Ecolinguistics and eco-cognition, in the context of discourse ecology and the interaction between discourse and cognition, embodies the concept of revisiting the discourse and cognition system to the natural ecosystem, developing into an ecologically beneficial model of discourse formulation and cognition mechanisms. Examining the CHD from the perspective of ecological dialectical cognition is conducive to providing an effective path for effectively spreading the Chinese harmony concept and dialectical approaches of cognition.

## Chinese Harmonious Philosophy

As discussed above, scholars had sought to classify ecolinguistics not as a science but as a philosophy related to cognition, which echoes the assertion of Chinese philosophy-based cognition, in order to find out more about the world and even may help to improve awareness and life.

### Brief Introduction

Harmonious discourse research comes from the urgent need to balance the critical discourse analysis (CDA) and positive discourse analysis (PDA) presenting themselves in solving the global ecological crisis respectively. The former criticizes those ecologically destructive discourses, and the latter seeks to find out various ecologically beneficial lexicons and expressions. The problem of the two lies in both presenting just one-sided research objectives, motivations, and methods. Either critical or positive stance is not sufficient for achieving real ecological harmony. That is why the harmonious discourse developed to balance the two is proposed, which meanwhile provides a pathway to amend some cognitive dissonance and deficiencies to a more abstract and higher one.

Critical discourse analysis is traditionally based on ideology and power relations. This ideology is explicitly or implicitly Marxist. Ecological discourse analysis (EDA) makes it quite different in that it founds itself on the preservation of life on earth and avoidance of suffering, especially avoidance of anthropocentric. For this purpose, the concept of “harmony” was proposed in discourse analysis. Thus, a new category which was “harmonious discourse” is presented.

As ecological meaning is a property of relations between living organisms and their environments (see Harvey, 2015; Trasmundi and Steffensen, 2016), ecologically harmonious discourse analysis should focus on this “property of relations.” The mentioned Chinese opposite formulations are taken as “relations,” and their unity covering the opposite two is taken as the “property,” used to improve originally opposite relationships or antagonistic ways of thinking to a more harmonious and unified level. Essentially, the Chinese DOU mechanism itself is the representation of ecological meaning. Therefore, it is generally seen as a multifaceted and problems-oriented research domain and communicative practices. Current global ecological crisis such as the COVID-19 virus, calls for a deeper probe into the theoretical basis and social praxis of “harmonious discourse” recently proposed in China, on a transdisciplinary scale of ecology, cognition, and discourses.

### Chinese Ecological Philosophy as the Basis of Harmonious Discourse

To achieve the first objective of this paper on a macro-level, to interpret the CHD in an ecological cognitive perspective, this paper first introduced the philosophy basis of both CHD and Chinese cognition mechanism, with the interaction and interchangeability of yin + yang Taoism as the essence, with the opposite expressions as the discourse structure.

#### Chinese Dialectical Harmonious Philosophy

The advanced search was carried out in WOS with the keyword of “ecological philosophy,” and the source category was the WOS core collection. The literature type of 1077 articles in related journals was searched, and 1,077 articles were analyzed by “keyword” with CiteSpace software. Time: 1976–2020.

As noted above, ecological philosophy presents colorful diversity in different academic fields. For example, the study of Naess (1995) used to propose a theory of “deep ecology” which advocates that the research of humanity is inseparable from that of nature, but it is still insufficient in forming the Marxist-based and Chinese-based ideology characterized by a kind of ecologically dialectical philosophy.

In this case, Chinese dialectical harmonious philosophy could be a valid candidate, with an emphasis on mediating various opposites into a deeper and higher sense of self and life, instead of following the mind/body dichotomy, providing a real place where Eastern philosophy, Western philosophy, and even quantum physics could meet one another.

Chinese ecological philosophy based on the tradition of Chinese culture and history has always been developing metaphysical foundations for establishing and improving harmonious social relationships. “Harmony” is widely acknowledged as the main concept in ecologically beneficial philosophy and discourse, aiming to establish and maintain a harmonious relationship between the environment and human beings. It has been already illustrated in Chinese CTM around which harmonious discourse revolves its main concerns, characterized by thoughtfully subtle implicitness and opposite-unity. Such a deliberate style of discourse is regarded as a deciding factor in keeping harmonious relationships.

#### The Nexus of Chinese Harmonious Philosophy and Dialectical Cognition

Chinese Taoism has been coherently taking the world as a whole for thousands of years, and the wholeness contains yin and yang sides. One Yin and one Yang are called Taoism, so as to keep the permanent energy conservation (能量守恒). Taoism has always had its role in guiding Chinese thinking, cognition, communication, education, and maxims in life.

**FIGURE 3:**
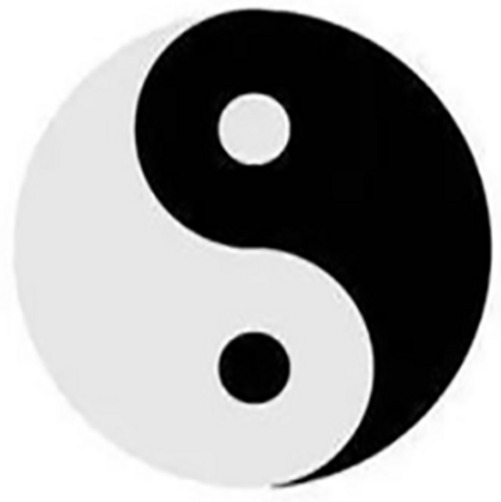
The Interaction and Interchangeability of Dialectical yin + yang Taiji.

As mentioned in Figure 3, according to Chinese cognition, everything has two sides which are yin and yang, and yin + yang constitute a state of Taiji. Yin and yang have been always interacting and interchanging in certain conditions, to permanently keep the life and growth in nature. Taoism Taiji is the quintessence of nowadays academic term “interaction,” seeking for developing a new understanding of ecologically beneficial ethics by balancing the two opposite sides into a harmonious state.

The core feature of such a dialectical interactive and interchangeable yin + yang model is that the understanding of yin only through contrasting with yang, and vice versa. These are principles of the whole universe and whole nature. Yin + yang by itself is a dialectical way of thinking, the process of which is through finding the common property of the two opposite concepts to achieve a subtly unified understanding of some ecological principles about life and behaviors. In certain conditions, yin could be converted into yang, and vice versa. Therefore, there always exist Chinese sayings “否极泰来” (when misfortune reaches its limit, good fortune is at hand), “塞翁失马焉知非福” (an old frontiersman loses his horse–a blessing in disguise).

Such yin + yang model constitutes the traditional Chinese philosophy and provides the formulation mode of CHD. The cognitive mechanism of these opposite two is through the process of “dialectical opposite-unity” to achieve the ultimate harmony, by way of “property” induction, refinement, and extraction. On this level, yin and yang are the two sides of a coin, and the two opposites have to be merged into a more abstract property covering the sense of the two, generating a subtle unified understanding in the form of an emergent property. Thus, CHD analysis is first based on and characterized by an ecologically beneficial philosophy with yin + yang dialectical unity, in order not only to superficially present the two extreme possibilities by words but also provide a way of balancing the yin and yang to achieve a more harmonious state of mind.

That is, ecological cognition is balanced and unified by the pros and cons of the same speech event or phenomenon to achieve Chinese Taoism with abstract yin + yang property, so as to realize the typical Chinese natural law of “Tao follows nature” (道法自然) and “harmony between man and nature” (天人合一).

#### Chinese Dialectical Philosophy Integrating With Other Disciplines

Similar to Taoism Taiji, nowadays quantum physics featured by its overthrowing the original dualism, reductionism, realism, and locality view of the world, presents a many-dimensional world and possible world view, thus reality being a higher-dimensional space, and the world view is changed thereby. Quantum thoughts hold that there existing two opposite beliefs in quantum entanglement of oneself which addresses the fundamental nature of reality, and the mind simultaneously accepting both of them, integrating them into a “Chinese yin + yang Taiji.”

From the above, Chinese ancient scholars, ecolinguists and quantum physicists have proposed that human beings have been cooperatively creating their “reality” with the interaction between natural ecology and social ecology, between the opposites into a dialectical unity. In this aspect, the research of CHD and its opposite-unity cognition actually offers new insights into the role of human minds and their quantum entanglement to the rest of the world. Chinese Taoism has some common maxims with twenty-first century quantum physics, integrating to promote researchers to re-define the key notions in linguistics and cognition.

Historically, Chinese Confucianists have always been attaching importance to “benevolence” to other human beings and nature. The law of Confucianism proposes to eliminate ethical evil and promote good and seek to establish and maintain social stability, harmony, and order. As a breakthrough of Chinese Philosophy in governing the country, Chinese Taoism seeks a deeper sense of “eternal rules” of dealing with the world. The representative scholar Lao Zi governs the country with “Taoism” in order to achieve ecological harmony and equality. Chinese Mohism takes “love and mutual benefit” as the ethical principle, with “salvation” as the core. It advocates that humans, nature, and society are inseparable wholeness, and develops the traditional dialectical “argumentation” of epistemology.

The above Chinese eco-cognition based on ecological philosophy is thought to be in the holism of CTM, with particular emphasis on harmony between humans, nature, and society. Thus, harmonious discourse and eco-cognition fall within a unified ecological worldview.

Chinese dialectical philosophy integrating with quantum physics evoke a revolution of ecological philosophy in unifying world views, values, ethics, and other concepts and ideologies into a concept of “Harmonious Coexistence” proposed by Chinese scholars, to make a Chinese contribution to the global ecology.

## Chinese Dialectical Cognition as the Mechanism of Harmonious Discourse

To achieve the second objective of this paper: on a meso-level, the illustration of the origin, definition, characteristics, and goals of a newly born ecological cognition in a newly born CHD, this section is to introduce the terminology of ecological cognition and its fundamental role in harmonious discourse.

### The Dialectical Opposite-Unity of Ecological Cognition

As to the origin of ecological cognition (eco-cognition), Chinese cognition characterized by DOU is generally deemed as a representation and iconicity to Chinese philosophy (相由心生), they have been usually probed interdependently, because of their common “harmony” perspective and assertion. So ecological cognition in China just means the harmony-oriented and Chinese philosophy-based mechanism of recognizing the world, to develop the ecologically beneficial approaches of constructing cognition models.

The common ground for the discussions of Chinese traditional dialectical cognition as the basis, mechanism, and process of harmonious discourse analysis is an attempt to conceptualize dialectical cognition as a kind of ecologically beneficial cognition with harmony as the core and main concern.

By virtue of a lack of systematic theories and methods in ecolinguistics, it is rational and necessary to integrate other theories, such as cognitive linguistics and philosophy, among others, to make multidisciplinary ecological discourse research. That is the reason for this paper taking ecological cognition and ecological philosophy as the supplement for ecological discourse study. As ecolinguistics is previously seen as a kind of hidden ideology study for protecting life, it has been developing an awareness of the interdependence among things and ideas, which could be further interpreted by Chinese yin + yang interaction and interchangeability.

As to the rationality of the concept of “dialectical” cognition, the Odense school bases its ecolinguistics, explanatory models, on both Marxist and non-Western models of dialectics, e.g., Buddhist philosophy (Bang and Døør, 2007, p. 37–42). They openly admit that Eastern philosophical and religious traditions contain useful information and tools for modern cognitive sciences. The term “dialectics” comes from a traditional and typical Chinese way of thinking, that is DOU, usually used to solve the cognitive dissonance about the deliberate opposite expressions and concepts to achieve a balanced harmony and of improving cognitive competence. Therefore, dialectical opposite unity is both a discourse style and a cognitive mechanism, to provide ecological benefit to all life through the state of harmony.

Thus, mind, ecology, and discourse constitute a necessary three-dimensional interactive unity. The process to develop the dialectical cognition is through the two opposite expressions or concepts, that is yin + yang, to acquire a more highly abstract and subtle property covering the two, establishing a “yin + yang → unity” cognitive mechanism, having its role in developing a harmonious state both in mind and discourses, contributing to an ecologically beneficial cognition construction, termed as “ecological cognition” (eco-cognition).

### The Rationality of Using Eco-Cognition in Harmonious Discourse Study

On the one hand, nowadays cognitive theories attach too much significance to the basic cognitive mechanisms such as image schema, metaphor, metonymy, and cognitive grammar, etc., resulting in some biased views of taking cognition as an unconscious process. Unconscious perspectives are destined to give rise to some cognition deficiencies, muddying the water of consciousness study, preventing the insight into higher cognition from being specified.

These deficiencies have been noticeably found out in two aspects: first, some scholars are programmed to take either a positive or critical approach as the unilateral final solution to the ecological crisis, especially in EDA, without the consideration of balancing and integrating the two within “harmony.” Such a phenomenon fully implicates that various cognition deficiencies negatively affect the human mind and behavior. Second, few people are capable of merging the two opposite concepts into one more abstract and subtle covering property, due to their habitually unconscious intention of trying to save their cognitive efforts. So they just understand the discourse meaning as they are, incapable of consciously analyzing, inducing, extracting, and refining the possible emergent property covering the two opposites, fully manifesting the cognition unconsciousness. Chinese DOU could be a candidate to solve the above problems.

Meanwhile, nowadays physical Quantum theories attach an increasing significance to consciousness, studied within the non-matter, energetic framework, echoing to Chinese subtle and abstract property covering the opposites for meaning unity. And Sills and Lown (2008) use an innovative and paradigm-crossing term from Buddhism namely, “the mind-body,” which is related to the human as the cognitive-physiological whole. These two schools believe that consciousness cannot be found totally in the physiological brain, it is announced by the integration of the ecological whole world. All of the above intend to prove that it is time for cognitive functions of humans to generate epistemological shifts and turns from the original ontology to a holistic underlying, superordinate, subtle, and abstract property, refining or extracting the identity (同一) or unity (统一) about cognitive construal toward the simultaneous happenings.

Besides, Anna Ba̧czkowska (2013) believes that “all living systems including man are phylogenetically (and ontogenetically) optimally designed and capable of harmonious and creative functioning within the reality they are functioning in,” according to which “harmony” is equipped by its distinctive “holistic and conscious” function in construing and constructing reality. Hence, the establishment of ecological cognition mechanism is effectively committed to solving problems of cognition deficiency and cognition unconsciousness, promoting them into a higher cognition, with ecologically beneficial cognition (eco-cognition) as the initiator and harmonious discourse as the output-based on such a function.

To sum up, by virtue of the cognition deficiency and unconsciousness, and based on a holistic theory proposed by quantum and ecologists, the main function of eco-cognition is to meet the requirement of a new multidisciplinary scientific research as cognitive tools and methods to recalibrate the philosophical, cognitive and socio-cultural perspectives of ecological harmonious discourse, creating a new ecologically beneficial holistic cognitive mechanism by DOU process, proposing a new harmonious cognitive mechanism to deal with the ecological crisis.

Such a new interactive wholeness world view substitutes the dualism and reductionism of the classical science, and directly turning the linguistic study breaking through the ontology limit into an epistemological one, shifting the ideology, values, and behavior into a dialectical yin + yang Taiji spiral recycle, renovating the basic cognition to an improved and upgraded level, to altogether build a new model of reality.

## The Analysis of Chinese Harmonious Discourse by Ecological Cognition Mechanism

To accomplish the third objective of this paper which was on a micro-level, the explanation of CHD by Chinese “DOU” ecological cognitive mechanism, in order to solve some cognitive dissonance and lift the basic cognition onto a higher level, this section presents ecological cognitive DOU mechanism in CHD.

Chinese harmonious discourse is characterized by the opposite expressions with reversible order, but with identical meaning characterized by property unity. For example, “否极泰来” (when misfortune reaches its limit, good fortune is at hand), the reversible order “泰极否来” still tells the same law and property, representing the same sense with “否极泰来,” with the meaning of everything going to the opposite if it reaches the extreme, based on the opposite-unity mechanism. Thus, the Chinese mediocre spirit is proved to be an acceptable life state to achieve real ecological harmony.

The effectiveness of the above ecological cognitive mechanism lies in its operational principle: opposites in expression(formulation) and unity in conceptualization by an emergent property. This is because Chinese discourses universally function as an initiator of consciousness toward some particular states, practices, and principles about life, ethics, values, thoughts, and behaviors, advocated by Taoism, Confucianism, or Mohism. In the process of pursuing the promotion of human nature, thoughts, behaviors, and ethics, the conscious process of induction, deduction, refinement, and extraction of property are habitually cultivated. Chinese have been successively and conventionally digging a deeper sense for discourses and speeches, whenever and wherever possible. Therefore, whether or not expression orders are reversible, they just tell the same story(sense) by retrieving the same subtle and abstract property as the higher-level cognition.

Deep ecologists insist that their philosophy is not a branch of environmental ethics, but something “deeper,” but the deeper aspects are not clarified. That is where the above Chinese CTM-based DOU philosophy functions. In the new context of quantum holistic worldview and Chinese philosophical wholeness combined, discourse analysis and their corresponding cognitive process are to be regarded as a mutually supported and mutually converted Taiji yin + yang or cause + effect perpetual recycle. Therefore, CHD is different from the general discourse studies which primarily focus on a linguistic level.

### The Etymology of Chinese Harmonious Discourse

Chinese harmonious discourse is a deliberate balance between critical and positive discourse biases, proposing the most important analyzing factor in ecological discourse, which is “harmony.” There exist two research tendencies in discourse studies seeking to solve the ecological crisis: CDA and PDA, the former is “an approach to language study which theorizes the instrumentality of language in creating and sustaining power and inequality in social actions, identities and relations” (Hart et al., 2020, p. 1). Several critical schools of CDA can be identified, characterized mainly by the theoretical and methodological frameworks that underpin their analyses (Hart and Cap, 2014; Wodak and Meyer, 2016).

Most ecological scholars hold a critical view toward the destructive discourse resulting in the ecology crisis. In order to reduce the number of destructive discourses and to increase that of positive discourses to achieve ecological harmony, scholars have been developing PDA. “A positive style of discourse analysis that focuses on hope and change, by way of complementing the deconstructive exposé associated with critical discourse analysis” (Martin, 1999, p. 29). To accomplish the positive purpose, scholars focus on the study of words correctness (Dunayer, 2001; Schultz, 2001), language use, framework construction (Macgilchrist, 2007), and storytelling (Robertson, 2014; Stibbe, 2015), in order to renovate discourses to enact a better world.

Through identifying the stereotypes of linguistic patterns in positive discourses, scholars intend to inspire respect and care for the natural world through finding out the positive features in discourse expressions. But PDA is also criticized that “One danger of (PDA), however, would be that of the enterprise turning into a form of propaganda on behalf of the status qu” (Flowerdew, 2008, p. 204).

The above two discourse studies fail to provide an appropriate solution to the nowadays ecological crisis, due to the lack of cognition in an ecological harmonious way. Critical and positive discourses, in their essence, are understood as both still holding the critical attitude in an ideal world, leading to their staying within a narrow research scope and only proposing ecological slogans such as peaceful co-existence and interdependence. Their just paying lip service to the wholeness world view and interactive model of ecolinguistics is insufficient in the micro and operational research of harmony philosophy, theory explanation, and social praxis, which is incapable of solving the ecology crisis caused by anthropocentric, economic development, and post-industrial civilization. CHD is expected to make some amendments to the above deficiencies, by the DOU mechanism.

The concept of CHD is the traditional native Chinese discourse theory (Huang, 2017; Wang, 2019; He, 2020, etc.), which could be used as an approach to balance and combine the critical and positive discourse, and to comprehensively demonstrate the whole dimensions of ecologically canonical events. Through evoking the deeper and higher cognition, we are in turn possible to develop and improve life insights of Taoism. The operational or specified way is that two opposite words (expressions)enact a conscious sense and connotation unity on the deep, subtle and superordinate level to acquire an abstract property.

Similar to the above, some scholars take consciousness as an emergent property of brains, which could be analyzed and interpreted by the Chinese eco-cognition mechanism, for it involves the conscious and deliberate cognition of the whole world in order to contribute to upgrading cognitive competence to a higher level.

### The Analysis of Chinese Harmonious Discourse by Ecological Cognition Mechanism

Both the “critical” and “positive” methods of discourse research all present a sort of one-sided bias, unqualified in establishing a dialectical wholeness in comprehending discourse, that is why CHD is presented and focused. The Chinese way of constructing a harmonious state, no matter in harmonious discourse or ecological cognition, is through the mechanism of the “property unity” covering the opposites, that is Yin + Yang → unity, integrating into a polished Taiji wholeness of construal.

As no corpus evidence could be provided due to the newness of CHD analysis, especially in the perspective of ecological cognition, this paper mainly adopts a theoretical analysis and interpretation. In general, the discourse realization process is represented by the Chinese convention of Yin + Yang, represented by Yin property + (matching, comparing, integrating) Yang property → Taoism (interaction + interchangeability) → dialectical emergent property(unity), both highlighting the concept of “harmony” as the deep-core sense in Chinese discourse. For example:

最明亮时总是最迷茫，最繁华时也是最悲凉。

                                           (林语堂《京华烟云》)

The brightest is always the most confused, the most prosperous is also the most sad.

(Lin Yutang’s smoke in Beijing)

The above “brightest” (yang) and “confused” (yin) and “prosperous” (yang) and “sad” (yin) constitute the opposite expression, deliberately juxtaposed to elicit different kinds of cognitive involvement (matching, comparing, and integrating), put together to acquire an emergent property (unity) or sense in the process of the deeper digging of their Taoism abstract property. Foregrounding two opposite words with the same importance certainly enacts a fundamental mediation to the cognitive dissonance from the opposites, effectively resulting in a third subtle, abstract, superordinate, emergent, balance-oriented property of a covering sense of these two words.

The instantiation of the operation of Taoism is clearly shown in the above utterance. Taoism, as a Taiji dialectical way of conscious thinking, is a complex thing, which cannot be decomposed into a simple group of simple components without destroying its essence. Thus, such unity is characterized by non-localized and *ad hoc* psychological quantum entanglement features, the understanding of which could only be the result of insight from dynamic nature and society. Put it in another word, the developing process of everything implicitly entails its opposite result, which reminds and implicates a necessity of cautious speech and behavior in a modest way, which is the optimal approach of achieving harmony.

Such a mechanism of cognition not only contributes to the ecological protection of life but also to the upgrading of intelligence. The research of newly born dialectical ecological cognition aims to consciously illustrate and evoke the harmony discourses, perspectives, concepts, and behaviors, which is capable of maintaining the ecology in a peaceful cycle.

Chinese dialectical harmonious way of discourse formulation has been devoting to the revision of the unilateral ideas about nature and society, acquiring a radical innovation in our reconstructing our recognition toward nature and reality, on a higher cognitive level. Chinese is always harmonious with every creature in the world, respecting nature and other human beings, which helps to solve the ecological crisis.

## Conclusion

Chinese harmonious discourse is possible to bring about an overt cognitive turn toward the inner insight, illocutionary sense, and emergent property, which has its role in altering the structure, strategy, and mechanism of discourse analysis, for the purpose of holding the whole ecology in a balanced, interactive and harmonious state, avoiding the ecological crisis brought about by all kinds of extremes, and adjust the behaviors to the changing environment of nature and society. The two opposites not only represent the interaction between static and dynamic states in life but also direct oneself to compare the two and probe deeper self and nature.

This harmony-oriented opposites-unity mechanism requires that the cognitive subjects mentally co-operate as a cognitive multiagent, evoking a more profound understanding toward ecological relationships, possible to enforce their cognitive agency and to improve their cognitive competence and thinking from an antagonistic way to harmonious way. The dialectical discourse and cognition mechanism are bound to stimulate a quite different mode of mental involvement, highlighting a state of unconscious property-extraction originating from a deliberate consciousness.

Dialectical ecological cognition is one of the most effective strategies for establishing linguistic and psychological interactivity. Foregrounding the process of intermingling opposite meanings within harmonious discourse mode may act as an innovative attempt to represent the fluidity of dialectical consciousness → unconsciousness → consciousness + unconsciousness intersections, contributing to “community of common destiny for all human beings” advocated by China.

## Author Contributions

The author confirms being the sole contributor of this work and has approved it for publication.

## Conflict of Interest

The author declares that the research was conducted in the absence of any commercial or financial relationships that could be construed as a potential conflict of interest.

## Publisher’s Note

All claims expressed in this article are solely those of the authors and do not necessarily represent those of their affiliated organizations, or those of the publisher, the editors and the reviewers. Any product that may be evaluated in this article, or claim that may be made by its manufacturer, is not guaranteed or endorsed by the publisher.
